# Engaging patients in balanced scorecard evaluation - An implication at Palestinian hospitals and recommendations for policy makers

**DOI:** 10.3389/fpubh.2022.1045512

**Published:** 2022-11-10

**Authors:** Faten Amer, Haroun Neiroukh, Saad Abuzahra, Yazan AlHabil, Mufeeda Afifi, Duha Shellah, Imre Boncz, Dóra Endrei

**Affiliations:** ^1^Doctoral School of Health Sciences, Faculty of Health Sciences, University of Pécs, Pécs, Hungary; ^2^Institute for Health Insurance, Faculty of Health Sciences, University of Pécs, Pécs, Hungary; ^3^School of Pharmacy, Faculty of Medicine and Health Sciences, An Najah National University, Nablus, Palestine; ^4^School of Medicine, Faculty of Medicine and Health Sciences, An Najah National University, Nablus, Palestine

**Keywords:** balanced scorecard, hospitals, patient-centered care, patient participation, quality of health care

## Abstract

**Introduction:**

A balanced scorecard (BSC) is a comprehensive performance evaluation (PE) tool. A recent review summarized that a balanced consideration of PE from six perspectives in hospitals must be considered: financial, customer, internal, external, knowledge and growth, and managerial. However, patients were rarely engaged in BSC implementations. This research aims to engage Palestinian patients in BSC implementation to develop recommendations for policy makers.

**Methodology:**

In this cross-sectional study, the BSC-PATIENT survey was distributed between January and October 2021. We evaluated patients' experiences and their attitudes toward BSC dimensions (BSCP ATT). The differences in evaluations based on admission status were analyzed using the Mann-Whitney U test. Causal relationships between patients' experiences and attitudes were analyzed using multiple linear regression. We tested the multicollinearity of the model. Path analysis was performed to understand the BSC strategic maps based on the Palestinian patients' evaluations.

**Results:**

Out of 1,000 surveys, 740 were retrieved. The mean scores for Palestinian patient experiences evaluation showed that the services experience factor had the highest score (87.7 ± 17.7), and the patient care experience factor had the lowest score (57 ± 34.5). Patient experiences collectively predicted 56.4% of the variance in the BSCP ATT. The experience factors of information (β = 0.400, *t* = 13.543, *P* < 0.001), patient care (β = 0.241, *t* = 8.061, *P* < 0.001), services (β = 0.176, *t* = 6.497, *P* < 0.001), and building (β = 0.177, *t* = 6.308, *P* < 0.001) had the highest impact on BSCP ATT. The price had only a weak negative influence (β = −0.051, *t* = −2.040, *P* = 0.042). Accessibility to hospitals did not have any impact on BSCP ATT. Significant differences between inpatient and outpatients' evaluations in regard to experiences related to patient care (*P* = 0.042), services (*P* < 0.001), accessibility (*P* < 0.001), and BSCP ATT (*P* = 0.003) were found.

**Conclusion:**

BSC-PATIENT successfully engaged patients in BSC PE at Palestinian hospitals. This research provides strong evidence for the impact of patients' information experience on their attitudes. Palestinian health policy makers must prioritize the design and delivery of patient education programs into their action plans and encourage a two-way information communication with patients. Strong evidence for patient care, services, and building experiences role in improving patients' attitudes was found. Managers should enhance patients' feedback and engagement culture in Palestinian hospitals.

## Introduction

### The Palestinian health care system

The health care system in the Occupied Palestinian Territories (OPT) is described to be fragile and incoherent (1, 2). This is due to the influence of the political and economic situation (1, 3–6). Furthermore, there are a variety of administrative hospital types in OPT. There are 28 public hospitals, 39 non-governmental organizations (NGOs) hospitals, 17 private hospitals, 2 military hospitals, and 1 United Nations Relief and Works Agency for Palestine Refugees in the Near East (UNRWA) hospital (7). As per the geographical location, 50 hospitals are in West Bank, 7 in eastern Jerusalem, and 30 in Gaza Strip (7). The bed percentage per administrative style is approximately 59%, 26% NGO, 14% private, and 1% UNRWA, whereas military hospitals are not yet operating (8).

### Performance evaluation (PE) of hospitals

The coronavirus (COVID-19) outbreak placed additional burdens on the world's health care system due to the limited capacity of hospital beds and the increased psychological stress of health care workers (HCWs) during the pandemic (9, 10). There is still a paucity of information that would assist health care managers and policymakers in enhancing the quality of health care delivery and learning for the future (11). Utilizing the key performance indicators (KPIs) in the health care system before the pandemic was important for a number of reasons. First, the patient and HCWs' satisfaction ratings were enhanced. Second, KPI utilization improves efficiency, effectiveness, and financial performance while adapting to new technologies and concepts. Third, using KPIs increases productivity and profitability (12–14). Last, keeping track of KPIs in a pandemic is particularly critical for health care organizations (HCOs), which may help identify areas that need immediate attention and strengthening (15).

#### Balanced scorecard

One of the strategic managerial tools that was implemented globally by many hospitals and utilized KPIs for the performance evaluation (PE) of HCOs is the balanced scorecard (BSC) (16). When Norton and Kaplan initially proposed the BSC in 1992, they incorporated four perspectives: financial (customer), internal process, knowledge, and growth (17). Other BSC implementations referred to the knowledge and growth perspective as the learning and development perspective (16). Additionally, some studies referred to the quality of care as a distinct perspective (18), whereas many of them referred to the quality as a part of the internal perspective (16). The external sustainability perspective was considered the fifth pillar to the BSC (19). However, our review of 36 BSC implementations indicated a need to add another two perspectives to the BSC design: the managerial and the external perspectives, which were frequently considered important by health care managers upon their implementation of BSC (16).

In the BSC second generation, researchers demonstrated the existence of causal links between the KPIs of these perspectives (20). These connections were known as the BSC strategic map. Next, the third generation was created, which comprised objectives and action plans for each KPI. Unlike other PE tools, most current PE models focus primarily on the internal viewpoint and do not address the other dimensions or perspectives that are as important. Two features distinguish BSC from other management methods. As a first component, it allows managers to concentrate on both financial and non-financial variables, providing a holistic approach to PE. Second, the BSC is more than just a planning or PE tool; it is also a strategic management tool. It assigns KPIs that are linked with the HCO strategy (20, 21). Other PE systems, such as total quality management (TQM), lack comparable comprehensiveness (22). Additionally, in our BSC systematic review, BSC implementation proved to positively improve the financial performance of HCOs (23). Furthermore, we found that BSC was beneficial in enhancing the patient satisfaction rate but mainly focused on measuring this aspect without engaging patients (16, 24).

#### Patient engagement in PE

Patient engagement has been an evolutionary topic in recent years (25, 26). Policy makers realized the necessity of having an evidence-based measure of patient engagement and capturing its influence (26). Engaging patients in health care is regarded globally as a crucial method for improving patients' adherence, clinical results, and satisfaction with the treatment they receive (27). A review of patient engagement during the COVID-19 era (25) found that there is a need for more original research on this topic during this era. It also found that engaging patients in policy-making decisions requires better attention. However, patient engagement is a complex and multifaceted experience (25, 26).

#### BSC-PATIENT instrument

The absence of patient engagement in BSC deployments and the lack of validated tools for this purpose (16, 28) prompted us to perform a systematic review to specify which dimensions were utilized in BSC implementations in general (16). Therefore, we developed a comprehensive instrument to engage patients in BSC implementations (BSC-PATIENT) based on the resulting dimensions (24). BSC-PATIENT was the first validated instrument to engage patients in the evaluation of hospitals based on BSC dimensions and perspectives. BSC-PATIENT was created to measure patients' experiences and attitudes toward hospitals based on these perspectives: the financial, internal, knowledge and growth, customer, external, and managerial perspectives. The BSC-PATIENT was also translated into Arabic and validated for use at Palestinian hospitals (24).

There is a difference between patients' experiences and attitudes. An event that was lived through is referred to as an experience (29). The health care service or treatment a patient gets at HCO is what shapes their experiences there. Experience perceptions are the consequence of being aware of events, objects, or relationships via the use of senses or observation (29). However, as a consequence of experiences, attitudes directly develop. There are three distinct types of attitudes, sometimes known as the ABCs of attitude (27, 30). First, the emotional aspect refers to how something, the individual, the situation, or the event makes somebody feel. The behavioral component examines how an individual's attitude affects their conduct. The cognitive component consists of an individual's ideas and opinions about the topic. Examples of attitude encompass image perception, satisfaction, and loyalty. These assessments are either favorable or unfavorable, but they may also be unclear or uncertain.

A review (31) explored how patient-reported experience measures are collected, communicated, and used to inform quality improvement across health care settings. This review found that patient experience data were most commonly collected through surveys and used to identify small areas that are not related to HCW behavior, such as admission processes and educational materials. This review also concluded that focusing on the evaluation of patients' experiences is still a relatively new field with a limited number of studies. On the other hand, the evaluation of patients' attitudes regarding HCOs is an essential duty for HCO management. Patients' attitudes, including their satisfaction, loyalty, and perceived image (PI), should be evaluated, and it must be determined what aspects of the patient's experience serve as predictors of those outcomes. Several studies have emphasized the significance of these various attitudes. First, patient attitudes are an important outcome measure since they represent the gold standard and a sign for gauging the quality of medical treatment (32–36). Second, patient attitudes provide assistance to HCO managers on how to determine which areas of patient experiences require improvement (32, 34–36). Third, patients' attitudes assist HCWs in knowing what they are doing properly or poorly, which improves patient care services (32, 35). Fourth, patients' satisfaction and trust may be predictive of whether they would adhere to and comply with HCWs' advice and treatment (32, 36). Thus, attitudes may function as mediators between patient experiences and the intended goal of their improved health status. Fifth, attitudes are associated with whether patients would return for treatment, follow up with their health care providers, or alter them (32, 35). This consideration can be important for private hospitals that aim to enhance their profits (33, 34). Last, HCO managers are usually concerned with how to make better resource allocations (34). All the previous factors reflect why it is important to determine which aspects of patients' experience impact their attitudes and to decide which of them deserves larger investment and attention. When taken into consideration as a whole, each of these factors will ultimately contribute to helping managers improve HCOs' PE.

In Palestine, researchers mainly focused solely on evaluating patient satisfaction. There is a lack of research in OPT that differentiates between patients' experiences and attitudes. This hindered Palestinian health policymakers from determining which patient experiences shape their attitudes. In addition, studies engaging patients in the PE of Palestinian hospitals are scarce. To the best of our knowledge, there has not yet been any research conducted in Palestine or anywhere else in the world that involves patients in BSC implementations using a survey (16, 24). This precluded top management from evaluating how various dimensions were performing based on the perceptions of their patients. In addition, we could not identify any published research employing BSC perspectives and dimensions to evaluate the PE of Palestinian hospitals, regardless of the data source or instrument used (16, 23, 28). In response to this gap, our study aims to (1) engage Palestinian patients in the assessment of Palestinian hospitals based on BSC perspectives and experiences and attitudes in their evaluations. Additionally, although limited research has analyzed the predictors for Palestinian patients' satisfaction, we could not find any study analyzing the predictors of dimensions, (2) determine the differences in patients' experiences and attitudes based on their admission status, and (3) assess which of the patients' experiences predict their BSCP ATT at Palestinian hospitals.

### Theoretical framework

**Figure 1:**
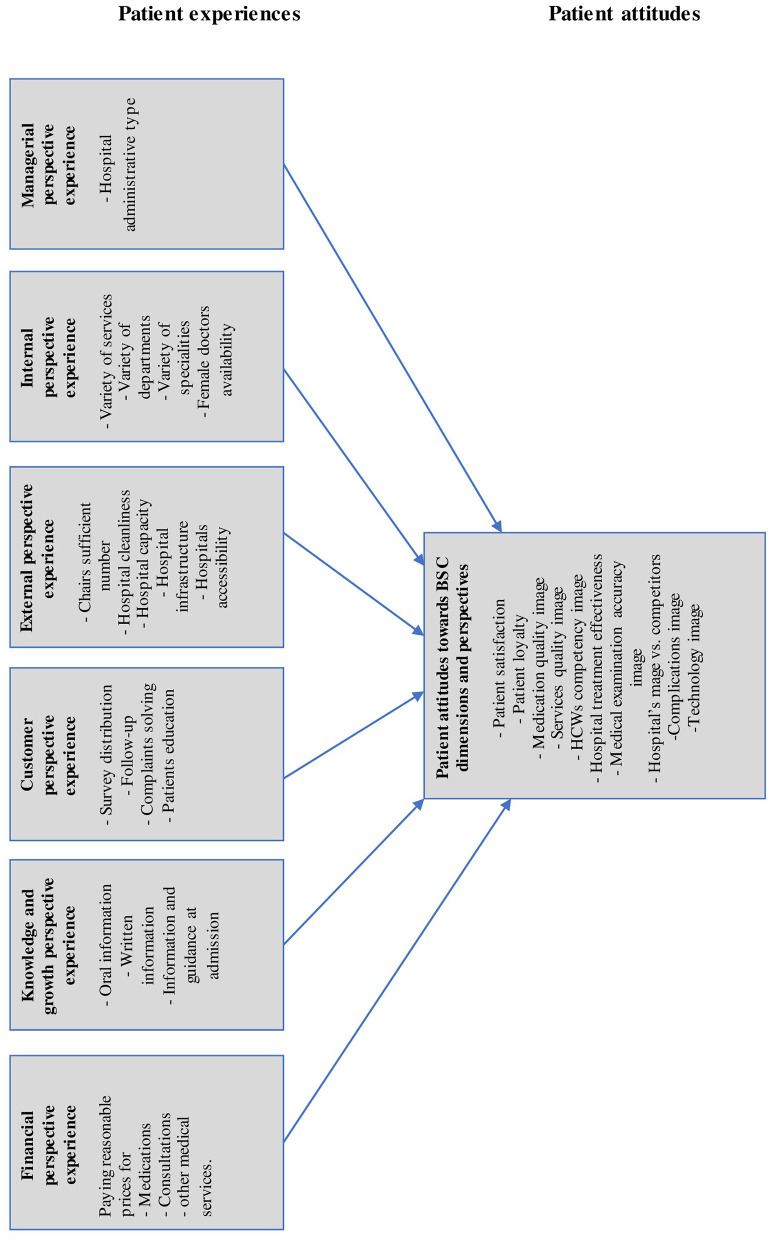
The theoretical framework for the impact of patients' experiences on their attitudes based on BSC.

Figure 1 represents the theoretical framework, which is built based on the development process of BSC-PATIENT (24).

## Methodology

### Study design and sample

This cross-sectional multisite design study is a part of a broad project (16, 23, 24, 37). This project aims to engage different stakeholders in the strategic development of Palestinian hospitals' performance via the use of BSC perspectives and dimensions. In this part, the focus is on engaging Palestinian patients in this aim. This research was reported in adherence with strengthening the reporting of observational studies in epidemiology (STROBE) checklist (38).

### Sample calculation

Since it was infeasible and geographically challenging to reach all hospitals in OPT, convenience sampling was used to choose 18 hospitals. Nevertheless, we took into consideration that our hospital sample has a variety of hospital sizes, locations, and administrative types. For that, the maximum variation sampling approach was utilized (7). Therefore, the number of hospitals and the number of beds in each administrative category and governorate were taken into account while selecting the sample of hospitals. Patient samples were also chosen conveniently. Patients who existed in the targeted departments at the time of the visit were asked if they were willing to participate in the research.

The sample size was determined using a Steven K. Thompson sample size calculation (39). The population volume in the Palestinian territories was considered the population size (7). Taking 0.5 as the estimated variability in the population, the margin of error was 0.05, and the *z* score was 1.96 at a 95% confidence interval (CI). Therefore, the number of patients required to conduct the study was 385. The authors were concerned about the poor survey response rate during the pandemic and opted to distribute 1,000 surveys. The patients were chosen because they were willing to take part in this study.

### Measures

In this research, we employed the Arabic version survey, which was designed and validated to engage patients in hospital PE at the BSC implementations (BSC-PATIENT) (24). The validation of the questionnaire revealed acceptable content validity, construct validity, convergent validity, discriminant validity, composite reliability, and interitem correlations.

The BSC-PATIENT validation revealed that the patient experiences loaded on seven factors: information experience (INFO EXR), price experience (PR EXR), patient experience (PT EXR), access experience (ACC EXR), service experience (SERV EXR), building environment experience (BUILENV EXR), and building capacity experience (BUILCAP EXR). The validation also revealed that all the Palestinian patients' attitudes loaded on one factor, BSCP ATT, except the patient image toward the technology (TECH IMAGE) and complications (COMP IMAGE), which loaded separately. In this research, we analyzed the effect of experience on BSCP ATT. We were not able to assess the influence of the management experience, which is represented by the administrative type in this study. This is because eight hospitals have permission constraints that prevent us from releasing any PE results connected to the hospitals' names or administrative types at the time being.

### Data collection

The first five authors were responsible for the data collection. A 3-h training session on BSC, data collecting procedures, and ethical considerations was provided by the lead author to the other authors prior to data collection. The coauthors were assigned tasks and hospitals based on where they lived: eastern Jerusalem and the northern, middle, and southern parts of the West Bank. The exclusion of the Gaza Strip was based on political and logistical reasons. In addition, five hospitals were excluded: two military hospitals that had not yet opened, one psychiatric hospital, and two rehabilitation hospitals.

Printed surveys were given to respondents between January and October of 2021 instead of delivering the questions through email to prevent non-response bias (40). To minimize the response bias (40), the “I don't know (neutral)” answer was introduced as an option because experiences and attitudes might sometimes be ambiguous (30). Second, the data collectors verified that the number of missing responses was reduced by examining the surveys upon recovery. In case of missing items, they drew the participant's attention to answer them. When inserting data, if any answers were discovered to be still lacking, they were recorded as I don't know.

A Palestinian patient who was at least 15 years old and of any gender was eligible for participation in the study. Outpatients should either have completed receiving medical care at the hospital being evaluated or should have received medical care at the evaluated hospital at least once in the past and returned there for additional treatment. Patients classified as inpatients must have been hospitalized for at least 1 day. The following departments were covered: pediatrics, gynecology, internal medicine, and surgery. Additionally, the emergency room was included. The patient companions were responsible for filling out the survey in the emergency room. One parent of each child was asked to complete the surveys in the pediatrics unit. The other surveys were completed by the patients themselves; in the instance that patients were unable to fill out the survey themselves, the data collector or a member of the patient's family read the survey to them and then completed the surveys based on the patient's responses. To make a distinction, a question was added that asked the responder if their answers were based on their personal experiences, the experiences of their family members, or the experiences of their friends.

### Statistical analysis

The data were coded by the first author. The normality of the data was tested by employing the Shapiro-Wilk test. Additionally, frequencies were calculated for the categorical patient-sociodemographic questions. The experiences and attitudes answers were converted to scale measures. For the 3-point Likert scale, “No” answers were coded as zero, “Yes” answers were coded as 100, and “I do not know” as 50. Frequencies for each question were calculated. Then, the mean score and standard deviation (SD) of each factor in both inpatient and outpatient categories were calculated based on the average of their underlying questions (41). Cronbach's alpha for each factor, subscale, and the scale were calculated after piloting. Based on this, the authors decided to combine the items of BUILENV EXR and BUILCAP EXR into one factor, BSC EXR, to raise the internal consistency. The remaining factors and items were kept the same.

To perform variance analysis for the factors based on admission status, we used the Mann-Whitney U test. Spearman correlation (*r*) was used to test the association strength between the independent factors or the dependent and independent factors. Then, *r* was described as negligible when *r* < 0.2, low (*r* = 0.2–0.49), moderate (*r* = 0.5–0.69), high (*r* = 0.7–0.85), or very high (*r* = 0.86–1.00). The cause-and-effect relationship was tested through a multiple linear regression with a *P*-value <0.05 for statistical significance and 95% CI. A path analysis is suggested to enhance the conceptual understanding and communication of regression results using an illustration (42). Therefore, we performed a path analysis of the dependent and independent variables to understand the strategic map of BSC from the patients' point of view. The residual plots were checked to test their normal distribution and linearity. Autocorrelation, which is also called serial correlation, was tested using the Durbin-Watson test (43). A value between 1.5 and 2.5 was considered in the acceptable range, as it indicates that the residuals have relative independence and that there is no serial correlation between them. Additionally, we examined our model for multicollinearity. Multicollinearity occurs when independent factors in a regression model are correlated, which is a problem because independent factors should be independent. Multicollinearity was detected if any of the following cutoff values were crossed (44, 45): (1) a Spearman correlation between factors was higher than 0.7, (2) a variance inflation factor (VIF) >10, (3) a condition index >30, (4) a variance decomposition proportion (VDP) for two or more predictors that were >0.8. All tests were performed using the Statistical Package for Social Sciences (SPSS) software version 21.0, except for the path analysis, for which we used IBM Amos Graphics software version 23.0. Additionally, the correlogram was prepared in R version (3.1.0).

### Ethical considerations

The Institutional Review Board (IRB) was issued on May 31 of 2020 by the Research and Ethics Committee at An Najah National University's Faculty of Medicine and Health Sciences with a reference code number (Mas, May/20/16). After that, we requested authorization to conduct the research at the public hospitals from the Palestinian Ministry of Health. Then, the request was submitted to every hospital individually, independent of its administrative type classification. Between June and December 2020, requests were to 15 hospitals on the West Bank and 3 hospitals in Jerusalem. Additionally, all of the patients provided written, informed consent to participate in the study that was in line with the Declaration of Helsinki ethical principles (46). Patients were assured of the confidentiality and anonymity of the data. Additionally, all of the patients were informed that taking part in the study was optional, so they could refuse to take part or leave the study at any time.

## Results

The response rate was 74%. The Shapiro-Wilk test result was significant, which revealed that the data were not normally distributed, so we decided to use non-parametric tests in the subsequent stages.

### Participant characteristics

The characteristics and sociodemographic characteristics of the patients are shown in Table 1. In our sample, there were two patients under 18 years old, and both were 17 years old. Consent to participate was obtained from their mother.

**Table 1:** Characteristics and sociodemographics of respondents (patients).

		**Number of patients**	**%**			**Number of patients**	**%**
		**(*N* = 740)**				**(*N* = 740)**	
Age (years)	Less than 20	63	8.5	Income (NIS)	Less than 1,000	195	26.4
	20–29	209	28.2		1,000–2,000	98	13.2
	30–39	208	28.1		2,001–3,000	152	20.5
	40–49	159	21.5		3,001–4,000	140	18.9
	50–59	71	9.6		More than 4,000	155	20.9
	60–69	24	3.2	Insurance type^#^	Public	492	66.5
	More than 70	6	0.8				
Gender	Females	325	43.9		Private	143	19.3
					UNRWA	63	8.5
	Males	415	56.1		No insurance	109	14.7
Highest degree	Elementary	85	11.5	Number of the current visit	First	227	30.7
	Secondary	217	29.3		Second	187	25.3
	Bachelor	366	49.5		Third	91	12.3
	Masters	63	8.5		Fourth	54	7.3
	PhD	9	1.2		Fifth	181	24.5
Working sector	Public	175	23.6	Admission status	Inpatients	350	47.3
	Private	183	24.7		Outpatients	390	52.7
	Free lancer	156	21.1	Respondent opinion is based on ^#^	Personal experience	570	77
	Retired	17	2.3				
	Unemployed	209	28.2		Family experience	306	41.4
					Friends experience	96	13

NIS, New Israeli Shekel; UNRWA, The United Nations Relief and Works Agency for Palestine Refugees in the Near East; NGO, non-governmental organization.

# Multiple response question.

### Descriptive analysis

The percentage of respondents' answers per question, as well as the means and SDs of the experience and attitudes factors, are shown in Table 2. The mean scores for patient experiences evaluation showed that the SEV EXR factor had the highest score (87.7 ± 17.7), and the PT EXR factor had the lowest score (57 ± 34.5). In total, the mean score of the Palestinian patient experiences and attitudes scale was (75.9 ± 17.2).

**Table 2:** Descriptive statistics of factors and underlying questions.

**Factors**		**Q no**.	**Question**	**Cronbach's alpha**		**Descriptive statistics**	
				**Factor**	**Subscale**	**No (%)**	**Yes^!^ (%)**	**Mean (±SD)**
IF (Experiences)	PTEXR	Q1	This hospital distributes surveys to assess my satisfaction before discharge	0.843	0.805	39.3	44.7	57 ± 34.5
		Q2	This hospital distributes surveys to assess my needs upon arrival to the hospital, admission, or during the stay			30.8	49.1	
		Q3	This hospital follows up with me after the discharge			36.1	42	
		Q4	My complaints are taken seriously into consideration and solved immediately at this hospital			25	41.5	
		Q5	Staff trained me on infection precaution measures such as hand hygiene, cough etiquette, isolation rational, personal protective equipment, etc.			33.8	58.1	
	PREXR	Q8″	I pay a reasonable price for the other medical services (laboratory, radiology, etc.) at this hospital	0.894″		29.5	70.5	86.4 ± 24.8
		Q9″	I pay a reasonable price for the medications at this hospital			5.7	93.4	
		Q10″	I pay a reasonable price for the medical consultation at this hospital			4.5	94.1	
	BUILENVEXR	Q11	There is a sufficient number of chairs in the waiting area	0.686^+^		39.9	55.2	70.8 ± 30.3
		Q12	The hospital has clean departments, corridors, rooms, bathrooms			13.1	82.2	
	BUILCAPEXR	Q13	The capacity of departments at this hospital including (ER, ICU, waiting room, etc.) is sufficient enough			30.1	60.5	
		Q14	This hospital has new building infrastructure (walls, ceiling, bathrooms, etc.)			18.6	70.7	
	ACCEXR	Q15	The accessibility to this hospital is easy by either public transportation or my car	0.821		24.6	70	71 ± 41.2
		Q16	The accessibility to this hospital in an emergency is easy			24.5	63.2	
	INFOEXR	Q17	Information provided to me to be used after discharge is sufficient (medication and side effects, health condition, etc.)	0.726		15	73.5	78.3 ± 30.7
		Q18	Oral and written information provided to me or my family during my hospital experience is sufficient			17.3	72.3	
		Q19	Information and guidance provided at admission or the first visit are sufficient			18.1	74.6	
	SERVEXR	Q20	Female doctors are available at this hospital	0.617		7.8	72.6	87.7 ± 17.7
		Q21	There are a variety of departments at this hospital			2.6	91.9	
		Q22	Services at night, vacations, and weekends are available at this hospital			8	79.2	
		Q23	There are a variety of specialties at this hospital			7.4	83.6	
DF (Attitudes)	BSCPATT	Q24	I will recommend this hospital to my family and friends		0.870	15.8	76.8	80.1 ± 25.1
		Q25	I believe I receive an accurate medical examination at this hospital			13.9	78.1	
		Q26	I believe this hospital offers me better treatment than the other Palestinian hospitals			22.4	59.9	
		Q27	My overall satisfaction with this hospital's performance is high	0.870		21.4	72	
		Q28	I believe this hospital has a high cure rate			9.2	72	
		Q29	I will choose this hospital again when I need a medical consultation			11.4	79.9	
		Q30	I believe the staff at this hospital are competent, knowledgeable, updated, and skilled			6.5	84.2	
		Q31	The services provided to me at this hospital have high quality			17.2	73.8	
		Q32	I believe the medications prescribed to me at this hospital have good quality and efficacy			9.6	71.8	

INFO EXR, information experience; PR EXR, price experience; PT EXR, patient-care experience; ACC EXR, access experience; SERV EXR, services experience; BUILENV EXR, building environment experience; BUILCAP EXR, building capacity experience; BSCP ATT, patients' attitudes toward balanced scorecard perspectives and dimensions.

″ Only patients who pay.

+ Items were merged to increase Cronbach's alpha.

! The rest answered "I do not know".

### Variance analysis for patient experience and attitude factors based on admission status

The variance analysis showed significant mean rank differences between inpatients and outpatients at the PTEXR, ACCEXR, SERVEXR, and BSCPATT. In general, the mean rank evaluation for all factors was higher for the inpatients (see Table 3).

**Table 3:** Variance analysis of BSC-PATIENT based on admission status.

**Factors**		**Mean rank**		**Z score**	***P*-value**
		**Inpatients (*N* = 350)**	**Outpatients (*N* = 390)**		
IF	INFOEXR	373.12	368.15	−0.35	0.726
	PREXR	367.72	372.99	−0.42	0.675
	PTEXR	387.25	355.47	−2.04	0.042
	ACCEXR	396.54	347.13	−3.61	<0.001
	SERVEXR	399.77	344.23	−3.88	<0.001
	BUILEXR	373.49	367.81	−0.37	0.709
DF	BSCPATT	394.28	349.16	−2.94	0.003

INFO EXR, information experience; PR EXR, price experience; PT EXR, patient-care experience; ACC EXR, access experience; SERV EXR, services experience; BUILENV EXR, building environment experience; BUILCAP EXR, building capacity experience; BSCP ATT, patients' attitudes toward balanced scorecard perspectives and dimensions; IF, independent factors; DF, dependent factors.

### Correlations

**Figure 2:**
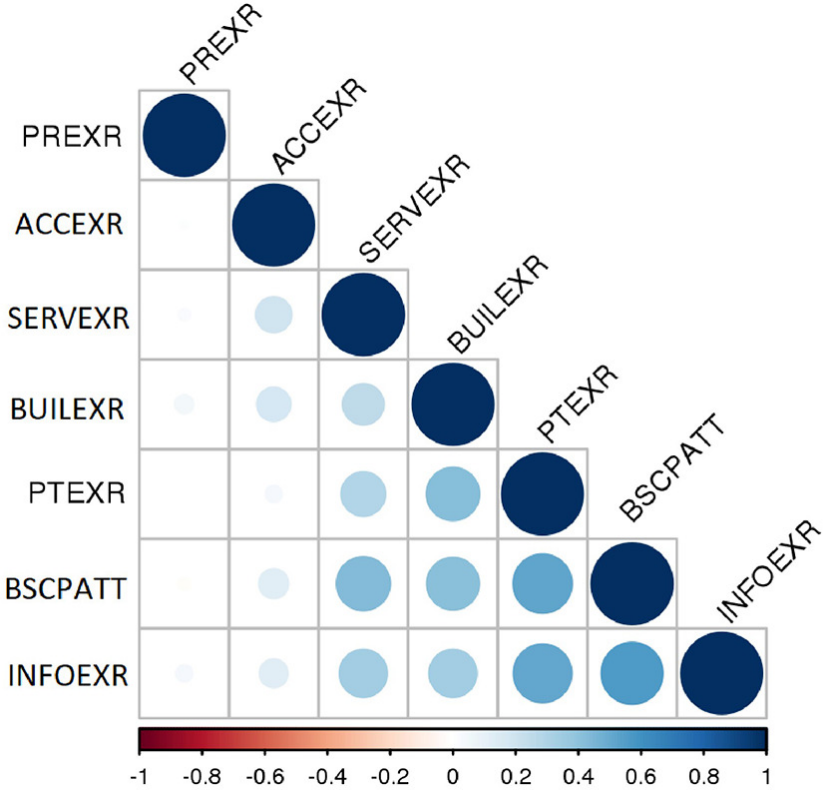
Spearman correlation (*r*) between BSC-PATIENT factors. *r* < 0.2, negligible; (*r* = 0.2–0.49), low; (*r* = 0.5–0.69), moderate; (*r* = 0.7–0.85), high; (*r* = 0.86–1.00), very high. PR EXR, price experience; ACC EXR, access experience; SERV EXR, services experience; BUIL EXR, building experience; PT EXR, patient-care experience; BSCP ATT, patients' attitudes toward balanced scorecard perspectives and dimensions; INFO EXR, information experience.

Spearman correlations between the experience factors were either negligible or weak. The correlation between INFOEXR and PTEXR was moderate. The correlations between the experience and attitude factors were either negligible or low, except between INFOEXR and BSCPATT, and PTEXR and BSCPATT were moderate. See the correlogram in Figure 2.

### The causal model

#### Multiple linear regression

The plot of residuals in SPSS revealed normal distribution and linearity. The Durbin-Watson value was 1.813. Multiple linear regression results showed that 56.4% of the variance in the patient attitude toward BSC perspectives and dimension evaluation can be collectively predicted by seven experience factor types [*F*_(6,733)_ = 160.522, *P* < 0.001]. Looking at the unique individual contributions of the predictors, the results show that INFO EXR (β = 0.400, *t* = 13.543, *P* < 0.001), PT EXR (β = 0.241, *t* = 8.061, *P* < 0.001), SERV EXR (β = 0.176, *t* = 6.497, *P* < 0.001), and BUIL EXR (β = 0.177, *t* = 6.308, *P* < 0.001) positively predicted BSCPATT. However, PREXR (β = −0.051, *t* = −2.040, *P* = 0.042) had a weak negative influence on BSCPATT. The ACCEXR effect was not significant (see Table 4).

**Table 4:** Evaluation of the causal effect between patients' experiences and their attitudes.

**Factor**	**Standardized coefficients**	***t***	***P*-values**	**CI**
	**β**			
INFOEXR	0.400	13.543	*P* < 0.001	[0.342, 0.458]
PREX	−0.050	−2.040	0.042	[−0.097, −0.002]
PTEXR	0.241	8.061	*P* <0.001	[0.182, 0.299]
ACCEXR	0.027	1.068	0.286	[−0.023, 0.076]
SERVEXR	0.176	6.497	*P* <0.001	[0.123, 0.229]
BUILEXR	0.177	6.308	*P* <0.001	[0.122, 0.232]
Model summary	*R*^2^ Adjusted = 0.564, *F*_(6.733)_ = 160.5, *P* <0.001	

BSCP ATT, patients' attitudes toward balanced scorecard perspectives and dimensions; INFO EXR, information experience; PR EXR, price experience; PT EXR, patient experience; ACC EXR, access experience; SERV EXR, services experience; BUIL EXR, building experience; CI, standardized confidence interval.

#### Path analysis

**Figure 3:**
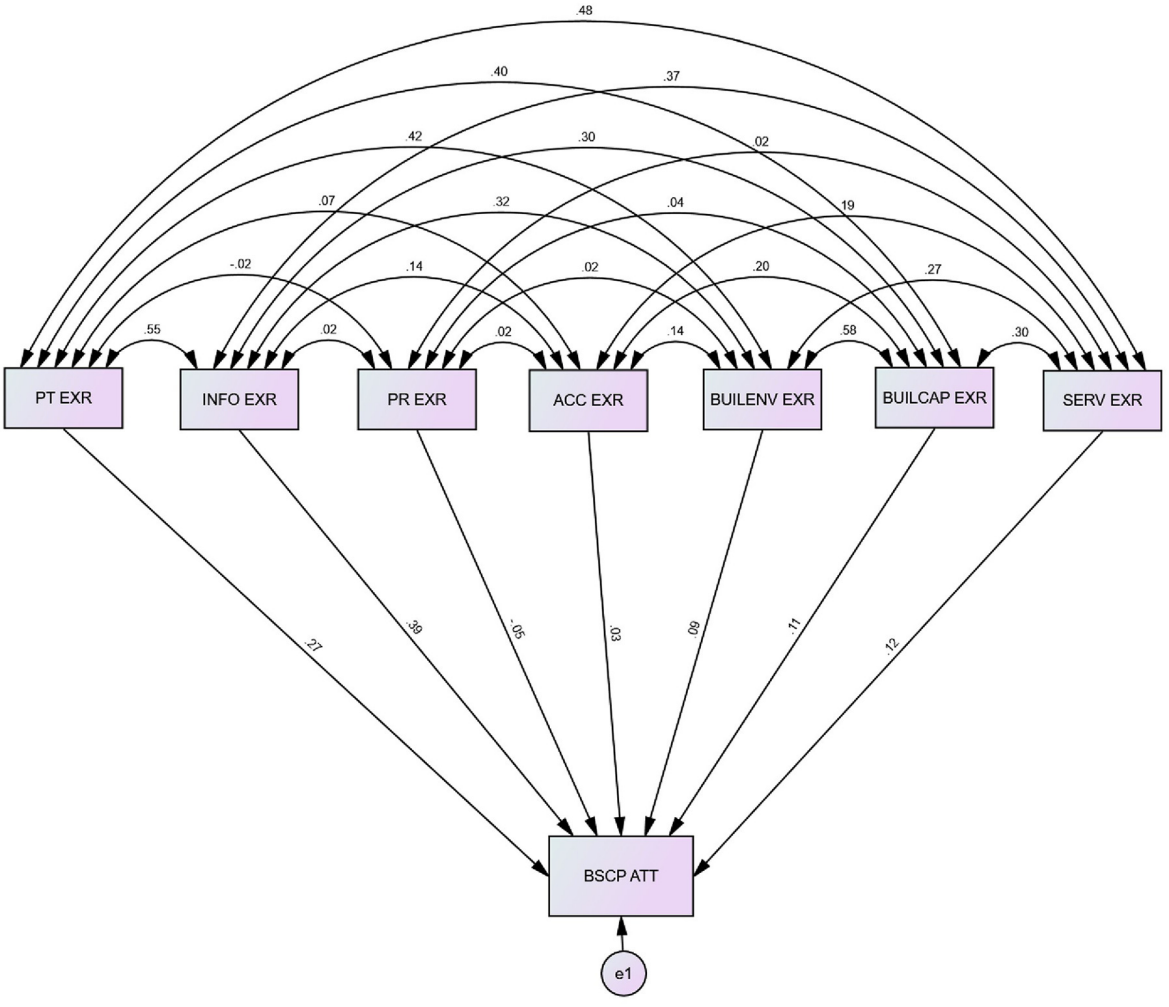
Strategic map of BSC based on Palestinian patients' evaluations using path analysis. The numbers on the straight lines reflect the standardized regression weights. The numbers on the curved lines represent the correlations between experience factors.

The strategic map from Palestinian patients' point of view using path analysis for the causal model is shown in Figure 3.

#### Multicollinearity analysis

The Spearman correlations between all independent factors were either negligible, low, or moderate in both groups. None were high or very high. The VIF values ranged between 1.005 and 1.510. The condition index ranged from 1.000 to 19.385. No VDPs for two predictors or more were above 0.8 for the same raw data.

## Discussion

### Discussion of the main results

In line with this paper's aim, we engaged Palestinian patients in the assessment of Palestinian hospitals based on BSC perspectives and dimensions using the BSC-PATIENT survey. The results revealed that an improvement in the performance of BSC dimensions is needed, mainly in the patient care, building, and information experiences, as their evaluation scored lower than 70%. Managerial focusing on improving the low-performing BSC dimensions will create the potential to improve the overall score of Palestinian patients' experiences and attitudes toward Palestinian hospital performance. Additionally, we assessed which of the patients' experiences at the Palestinian hospitals predicts their attitudes toward the BSC perspectives and dimensions of performance.

The causal model revealed acceptable autocorrelation and no multicollinearity. Analysis of the causal model revealed that the patient experiences that had the greatest positive impact on patients' attitudes toward the performance of BSC perspectives dimensions were information, patient care, and services. The building dimension also had a positive impact but to a lesser extent. The price had a mild negative impact. Accessibility to hospitals did not have any impact on patients' attitudes. These results were confirmed by both multiple linear regression and path analysis. To improve Palestinian patients' attitudes, these patients' experiences should be focused on. One explanation why the price did not play a significant role in Palestinian patients' attitudes is that only 14.7% of Palestinian patients do not have any type of medical insurance. Finally, there were significant differences between inpatients' and outpatients' evaluations regarding their experiences and attitudes at the Palestinian hospitals. Specifically, those experiences related to patient care, services, accessibility, and patient attitudes toward BSC perspectives and dimensions were lower for the outpatient group. Therefore, Palestinian hospital managers need to consider how to enhance these experiences and attitudes among outpatients.

### Comparison with BSC implementations worldwide

As previously stated, to our knowledge, no BSC study has been conducted in Palestinian hospitals. Additionally, in comparison with BSC implementations worldwide (16), only 22% of researchers included patients in the BSC. However, they mainly focused on patient satisfaction, and none used a survey to engage patients in BSC implementations (16, 24). This reflects the significance of BSC-PATIENT utilization and the uniqueness of this investigation.

### Comparison with other studies in OPT

A study (47) was conducted in one Palestinian city, Nablus, focused on measuring inpatient satisfaction and its predictors. In contrast to our study, no distinctions between patient experiences and attitudes were made. For example, the assessment of the information provided to patients' experience was mixed with the patient attitude toward the quality of treatment, accuracy of diagnosis, and attitude toward nursing staff training in one factor. This construct was referred to as the technical quality. Moreover, time spent and courtesy of staff experiences were mixed with patients' attitudes of trust and confidence of HCWs under one factor: interpersonal skill. The effect of factors on patient satisfaction was in the following order: room services, interpersonal skills, accessibility, and technical quality. This is different from our results sequence. A study of outpatients in an emergency department at the Gaza strip (48) performed only the measurement evaluation and analyzed the effect of sociodemographic factors on patient satisfaction. However, we did not analyze the causal relationships of experiences on attitudes. However, researchers have referred to the importance of not only conducting PEs but also understanding the causal relationships among the variables (49). Compared with a study in the city of Jerusalem (50), which measured the causal relationships of experiences on patient satisfaction. It found that four factors have an effect on this outcome: time and access, physical environment in health care centers, cost and health insurance, and comprehensiveness and quality of health care. Compared with our sample, all the studies we found in OPT were either performed in one city, assessed a selective department, or assessed only one hospital administrative type. Our study sample was the first to cover the north, middle, and west areas of West Bank as well as Eastern Jerusalem, which represents most of the OPT areas. In addition, this study is the first to cover all administrative types of hospitals in OPT and a variety of patient characteristics and departments. Additionally, there is a scarcity of studies performing such a purpose during the pandemic.

### Strengths and limitations

This research has several strengths. First, this is the first study that engages patients in the evaluation of hospitals based on BSC dimensions. Second, this is also the first study to utilize the BSC-PATIENT survey to assess which of the patients' experiences predict their attitudes toward the BSC perspectives and dimensions of performance. This utilization will allow managers to highlight the gaps in their performance based on patients' observations and opinions, which will eventually contribute to enhancing the PE of Palestinian hospitals. Third, this is the first study to assess the differences between patients' experiences and attitudes based on their admission status in Palestine. Fourth, this is one of the few available research projects that aims to engage patients in the PE of Palestinian hospitals. Most available studies focused mainly on measuring patient satisfaction and lacked the distinguishing between their experiences and attitudes in the scale used. Consequently, this research will not only assess patient satisfaction but also measure other patients' attitudes, as well as a better understanding of these attitudes' predictors. Fifth, to our knowledge, this is the first study to consider the PE of Palestinian hospitals during the pandemic era. Finally, this is one of the largest research projects involving the PE of Palestinian hospitals to date. As in this research, we considered including 30% of the Palestinian hospitals. Additionally, we included all the varieties of hospitals in our sample: the area, hospital administration type, hospital size, and hospital accreditation status. In addition, the varieties in patients' types were recorded based on their admission status, departments, area, and insurance type. This will lead to a better generalizability of the study results among the rest of Palestinian hospitals and patients and a better comparability possibility. However, we did not include these variables in our statistical analysis in this study due to hospital approval constraints. Future research is still needed to assess the impact of hospital and patient characteristics on patients' experiences and attitudes after obtaining the permission of eight hospitals to publish such analysis. Other limitations of this study are that although this instrument evaluates items such as patient education on infection control measures, it lacks COVID-19-specific questions. This is referred to the reason that this instrument was created before the COVID pandemic. Therefore, COVID-19-related items may be examined in future editions of the BSC-PATIENT instrument. Third, gauging patient experiences in the past may have included a recall bias. Finally, this research performs an evaluation for hospitals only from the patients' view. Research is needed to evaluate these hospitals based on BSC perspectives and dimensions from the provider's view, including managers and HCWs.

### Practical and theoretical implications

This research offers broad practical implications for Palestinian hospital managers, as they will be able to better focus on the following:

1. Developing a formal training plan for HCWs to improve the information provided to patients upon their admission and discharge, including oral and written information.
2. Investing in formal training to HCWs to improve patients' education, such as education on infection control measures.
3. Improving the receipt of information and feedback from Palestinian patients through the distribution of surveys. Additionally, serious consideration to solve patients' complaints is encouraged.
4. Increasing the variety of specialties and departments available at Palestinian hospitals, as well as the availability of medical services at nights, vacations, and weekends. Additionally, ensuring the availability of female doctors and nurses in all departments is a demand that can be referred to the Palestinian culture.
5. Improving the building dimensions, including the environment, such as the cleanliness, infrastructure, and capacity of departments. Many patients reported that the number of chairs in the waiting area had to be increased in the Palestinian hospitals.
6. Improving Palestinian outpatients' experiences related to patient care, services, and accessibility, as well as the outpatient attitudes toward BSC perspectives and dimensions.
7. Engaging Palestinian patients in future hospital PEs by utilizing the BSC-PATIENT instrument for such purposes. This should be carried out routinely to monitor the change and improvement in the quality of health services from patients' observations.

Additionally, this research has theoretical implications for future research:

1. Assessing the impact of hospital and patient characteristics on patients' experiences and attitudes.
2. Comparison between the PE based on the managerial evaluation and hospital records with other stakeholders' evaluations, such as patients and HCWs.

## Conclusion

In conclusion, in this research, it was possible to engage Palestinian patients in the assessment of Palestinian hospitals based on BSC perspectives and dimensions. The patient experiences that had the greatest positive impact on patients' attitudes toward BSC perspectives and performance dimensions were the information, patient care, services, and building dimensions. Hospital managers need to focus more on investing time and resources to improve the performance of these dimensions. Mainly, future emphasis on patient education programs and information exchange between HCWs and patients is essential. The price had only a mild negative impact. Accessibility to hospitals did not have any impact on patients' attitudes. Inpatient evaluations were better than outpatient evaluations in regard to experiences related to patient care, services, accessibility, and attitudes toward BSC perspectives and dimensions. Palestinian hospital managers need to focus on separate evaluations of patient experiences and attitudes to better understand the causal relationships among them. By doing so, they can better understand their patients and highlight the experiences that predict their attitudes toward their hospitals. This analysis will lead to customized action plans to improve these experiences. The BSC-PATIENT survey can help managers perform this purpose. However, patient feedback culture has to be encouraged in Palestinian hospitals. Additionally, using BSC-PATIENT should be combined with continuous follow-up for action plans and frequent measurements to monitor the effectiveness of action plans and hence to strategically improve hospitals' PEs.

## Data availability statement

The raw data supporting the conclusions of this article will be made available by the authors, upon reasonable request. Requests to access the datasets should be directed to amer.faten@etk.pte.hu.

## Ethics statement

The studies involving human participants were reviewed and approved by the Research and Ethics Committee at the Faculty of Medicine and Health Sciences at An-Najah National University with reference code number (Mas, May/20/16) on 31 May 2020. The patients/participants provided their written informed consent to participate in this study.

## Author contributions

This paper's conception was planned by FA. Additionally, FA, HN, SA, YA, and MA were responsible for obtaining hospital approval and data collection. FA was responsible for statistical analysis, interpretation of data, and writing the final draft. FA, HN, SA, YA, MA, DS, IB, and DE substantially revised the final manuscript draft. FA, HN, SA, YA, MA, DS, IB, and DE approved the submitted version and agreed to be personally accountable for the author's contributions and to ensure that the accuracy and integrity of any part of the work were appropriately investigated and resolved.

## Funding

This research was financed by the Thematic Excellence Program 2021 Health Sub-Programme of the Ministry for Innovation and Technology in Hungary, within the framework of the EGA-10 project of the University of Pécs and the National Laboratory on Human Reproduction (RRF-2.3.1-21-2022-00012) Program. Data collection expenses were personally funded by FA.

## Conflict of interest

The authors declare that the research was conducted in the absence of any commercial or financial relationships that could be construed as a potential conflict of interest.

## Publisher's note

All claims expressed in this article are solely those of the authors and do not necessarily represent those of their affiliated organizations, or those of the publisher, the editors and the reviewers. Any product that may be evaluated in this article, or claim that may be made by its manufacturer, is not guaranteed or endorsed by the publisher.
